# Diagnostic validity of the MINI-KID disorder classifications in specialized child and adolescent psychiatric outpatient clinics in Sweden

**DOI:** 10.1186/s12888-019-2121-8

**Published:** 2019-05-09

**Authors:** Camilla Högberg, Eva Billstedt, Caroline Björck, Per-Olof Björck, Stephan Ehlers, Lars-Henry Gustle, Clara Hellner, Henrik Höök, Eva Serlachius, Mats A. Svensson, Jan-Olov Larsson

**Affiliations:** 1 Child and Adolescent Psychiatry, Stockholm County Council, Stockholm, Sweden; 2 Institute of Neuroscience and Physiology, University of Gothenburg, Gothenburg, Sweden; 3 Function Allied Health professionals, Stockholm County Council, Karolinska University hospital, Solna, Sweden; 4 Child and Adolescent Psychiatry, Region of Skåne, Lund, Sweden; 5 Child and Adolescent Psychiatry, Sahlgrenska University Hospital, Gothenburg, Sweden; 6 Centre for Psychiatry Research, Department of Clinical Neuroscience, Karolinska Institutet & Stockholm Health Care Services, Stockholm County Council, Stockholm, Sweden; 7 Department of Women’s and Children’s Health, Karolinska Institutet, Widerströmska huset, Tomtebodavägen 18A, S-171 77 Stockholm, Sweden

**Keywords:** Validity, Standardized interview, Child and adolescent psychiatry, LEAD, MINI-KID

## Abstract

**Background:**

Missing diagnostic information often results poor accuracy of the clinical diagnostic decision process. The Mini International Neuropsychiatric Interview for Children and Adolescents (MINI-KID) is a short standardized diagnostic interview and covers a rather broad range of diagnoses applicable to children and adolescents. MINI-KID disorder classifications have shown test-retest reliability and validity comparable to other standardized diagnostic interviews and is claimed to be a useful tool for diagnostic screening in Child and Adolescent Psychiatric care. The concordance between the Swedish language version of the MINI-KID Interview and LEAD (Longitudinal, Expert, All Data) research diagnoses was studied in secondary child and adolescent psychiatric outpatient care.

**Methods:**

MINI-KID interviews were performed for 101 patients, boys *n* = 50, girls *n* = 51, aged 4 to 18 years. The duration of the interview was on average 46 min, the child/adolescent participating together with the parent(s) in most cases. The seven most prevalent diagnoses were included in the analyses.

**Results:**

The average overall percent agreement (OPA) between MINI-KID and LEAD was 79.5%, the average percent positive agreement (PPA) 35.4 and the average percent negative agreement (NPA) 92.7. OPA was highest for Obsessive-Compulsive Disorder (OCD) (0.89), Tic disorders (0.88) and Pervasive developmental disorders (0.81). There were similar results in diagnostic agreement comparing the two versions: the standard MINI-KID and MINI-KID for parents. The specific screening questions in MINI-KID resulted in additional preliminary diagnoses compared with the regular initial clinical assessment.

**Conclusions:**

Overall, there was an acceptable agreement between MINI-KID disorder classifications and research diagnoses according to LEAD. The standardized interview MINI-KID could be considered as a tool with the possibility to give valuable information in the diagnostic process in child and adolescent care which is similar to the setting in the present study.

## Background

Clinical interviews in Child and Adolescent Psychiatry obtain information about symptoms and impairment related to various disorders, however, missing diagnostic information often gives poor accuracy of the clinical diagnostic decision process [1, 2]. Missing diagnostic information could be caused by deciding on a diagnosis before collecting all relevant data and terminating the interview before exploring all alternatives [3]. One way to increase reliability is to evaluate a child’s mental health status using a standardized diagnostic interview. Consequently many interview instruments have been developed to evaluate the mental health of children and adolescents [4]. MINI-KID is included in a recent review that describes the characteristics of six published Structured Diagnostic Interviews (SDI), based on the Diagnostic and Statistical Manual of Mental Disorders (DSM) that are available to researchers and clinicians [5, 6].

MINI-KID is a short standardized diagnostic interview for DSM-IV [7] and the International Statistical Classification of Diseases and Related Health Problems - Tenth Revision (ICD-10) [8] psychiatric disorders in children and adolescents. MINI-KID is an extension of the adult version of the Mini-International Neuropsychiatric Interview (MINI) [9]. The instrument uses two to four screening questions for each disorder. If the screening questions are positively answered, additional symptom questions are given for the particular disorder. This is in contrast to an unstructured clinical interview running the risk of having to terminate the interview to early. The instrument can be administered by interviewing parent(s) and adolescents together or separately. There is also a newer version of MINI-KID adapted to DSM-5 [10] available (version7.0.2), not yet translated into Swedish. MINI-KID provides fewer disorders than the more comprehensive Schedule for Affective Disorders and Schizophrenia for School Aged Children-Present and Lifetime Version (K-SADS-PL) [11] but it covers a rather broad range of diagnoses applicable to children and adolescents and takes much less time to administer. The administration of the K-SADS-PL takes approximately 1.25 h for each parent and child (total time 3 h). MINI-KID has an administration time of 15 to 50 min [6]. MINI-KID disorder classifications have shown test-retest reliability and validity comparable to other standardized diagnostic interviews. Thus, MINI-KID is claimed to be a useful tool for diagnostic screening in Child and Adolescent Psychiatry [5, 12, 13].

The reliability of the MINI-KID disorder classifications and its validity has been compared with K-SADS-PL, using it as the reference or the gold standard. The authors concluded that MINI-KID generates reliable and valid diagnoses in a much shorter time than K-SADS [11]. In a recent Swedish study, the K-SADS showed very good validity for most major child psychiatric diagnoses [14]. However, in that particular study the LEAD assessment included all information from the K-SADS interviews. The administration and interpretation of K-SADS require clinical training and expertise and should be administered by a trained interviewer. Thus, the instrument may not be suitable for use in a clinical setting by an untrained clinician or as the only tool for validation of structured diagnostic interviews. According to Brooks et al., in a review of commonly utilized instruments concerning diagnosis of depressive disorders “Given its extensive demands on time and expertise, and the fact that objective criteria are provided alongside each grade on its 3-point symptom coding scales, its reliability is not particularly impressive” [15].

A study of the MINI-KID estimated the test–retest reliability for parent-, children- and adolescents-assessed DSM–IV disorders in clinic and general population samples. It compared the factor structure of internalizing and externalizing disorder constructs assessed by the MINI-KID and the Brief Child and Family Phone Interview (BCFPI) as an independent measure of disorder [12]. The study showed estimates of test–retest reliability and validity comparable to other standardized diagnostic interviews. The authors conclude that “These findings, in addition to the brevity and low administration cost, make the MINI-KID a good candidate for use in epidemiological research and clinical practice.”

However, a recent comprehensive review by Duncan et al. of published evidence on the test–retest reliability of SDIs raises important questions about the overall usefulness of SDI:s in both clinical and research settings [16]. The pooled test-retest reliability was moderate at Kappa 0.58 and between study heterogeneity was substantial. In addition, previous research has shown low agreement between clinician-generated diagnoses and those from structured diagnostic interviews [2].

Most prior studies of agreement have not used research diagnoses based on the gold standard methods. One way is to use expert clinical judgements based on information from medical records, to generate best estimate diagnoses in order to come close to the gold standard. Such research diagnoses are operationalized by LEAD (Longitudinal, Expert, All Data) suggested by Spitzer in 1983 [17]. Spitzer suggests the use of multiple sources of information and monitoring of the patient’s condition and diagnosis over time. In the absence of the established gold standard, LEAD has been widely used in psychopathology research for studying validity of diagnostic procedures [2, 14, 18–27]. The MINI interview for adults has been validated using expert’s diagnoses [28]. To the best of our knowledge, MINI-KID has not been validated previously using expert’s diagnoses and the LEAD procedure.

## Aim

The main goal of the present study was to analyze the concordance between the Swedish language version of the MINI-KID Interview and LEAD (Longitudinal, Expert, All Data) research diagnoses was studied in secondary child and adolescent psychiatric outpatient care. We also studied the relationship between MINI-KID disorder classifications and the diagnoses from the regular initial clinical assessment, expecting a low agreement mainly driven by missing information in the regular initial clinical assessment resulting in lower number of diagnoses compared with the MINI-KID disorder classifications. An additional aim was to compare the two versions of MINI-KID; the standard MINI-KID for children/young persons and the newer version MINI-KID for parents. The MINI-KID disorder classification derived from the two versions were compared with the LEAD assessments.

## Methods

This is a collaboration project between researchers and clinicians in three parts of Sweden: Stockholm, Gothenburg and the region of Skåne. In Sweden, the child and adolescent mental health services are divided into 1) the primary mental health care with physicians not licensed as specialists in child and adolescent mental disorders and psychologists and 2) the specialized mental health care, secondary level, with staff working in multidisciplinary teams (licensed specialists, i.e. psychiatrics/child psychiatrists, residents, and psychologists, counselors, registered nurses, occupational therapists and others). The present study was conducted in outpatient clinics in group 2. The service is free of charge for the families and part of the general healthcare system in Sweden.

The target population was all new referrals to five outpatient clinics: three in the south-east region of Stockholm (Ektorp, Globen och Farsta), one in the region of Skåne (Lund/Eslöv/Landskrona) and one in Gothenburg (Kungshöjd). Data collection was performed during the years 2013–2016. The exclusion criteria were: 1) recent contact with child and adolescent psychiatry (< 1 year), 2) not spoken Swedish (need of interpreter), 3) known mental retardation (IQ < 70). According to the protocol, the MINI-KID interview in the research project should be conducted after the first regular visit to the clinic, but not more than 6 weeks after the first regular visit. The plan was to use consecutive selection. The study was performed in the regular clinical units and the project coordinator (first author CH) informed the staff and regularly visited the clinics. In spite of these efforts, the study took longer time to perform than we originally expected. The main reason was that a very small proportion of the families in the target population accepted to participate in the study. Another reason was that many families were not asked by the staff to participate. For example, 53 patients were included in the study from the three clinics in Stockholm though having a total of 11,409 new referrals during 2013–2016. There were also logistical difficulties performing the MINI-KID interviews within the time limit (not more than 6 weeks after the first regular visit to the outpatient unit) for the families who were willing to participate in the study. Thus, the sampling procedure resulted in a sample of convenience. Demographics of the study group are presented in the first paragraph of the Results section.

### Procedure

#### Initial clinical assessment

The initial clincial assessment and preliminary / tentative diagnosis was made by the patient’s regular clinician at the unit in accordance with clinical practice for outpatient care within each region. The clinician performing the initial clincial assessment is either a resident in child and adolescent psychiatry, psychologist, counselor or a registered nurse. In the present study “initial” was defined as all visits and assessements made during 6 weeks after the first regular visit to the outpatient unit. During the first 6 weeks, the average number of visits was 1.9, median 1 SD 1.3, min 1, max 8. The clinician-generated diagnoses were mainly generated through unstructured interviews, that is not using SDI:s; in a few cases short questionnaires were used, for example regarding symptoms of depression.

#### Mini-kid

MINI-KID interview in the research project: was conducted after the first regular visit to the unit, but not more than 6 weeks after the first regular visit. The clinician at the unit performing the clinical assessment did not receive any information about the results of the research MINI-KID interview. The outcome of the MINI-KID interview was not returned directly to the patient/parents and not included in the medical record. However, the patients/parents were offered to be informed of the results of the MINI-KID interview after the LEAD assessment was completed. The parents were not reimbursed for their time but the children were given movie tickets.

The interviewers were physicians (*n* = 2), psychologists (8), social workers (5) and registered nurses (2). The number of interviewers in the three sites were: Stockholm *n* = 11, Gothenburg *n* = 4, and Skåne *n* = 2. They underwent a one-day project-specific education followed by training interviews. Each person conducted 3 interviews to be certified by the MIN-KID trainers. The interviewers who were approved were offered supervision when needed and participated in a follow-up of the training day after 6 months. The MINI-KID interviews were video recorded and a random selection was examined to check for the interviewers’ compliance with the method.

It is a brief semi-structured interview for children between the ages of 4 and 17, developed in collaboration between psychiatrists / clinicians in the USA and Europe for diagnoses [5]. MINI-KID includes modules covering depressive disorders, suicidality, bipolar disorders, anxiety disorders, obsessive compulsive disorder, posttraumatic stress disorder, alcohol abuse, substance abuse, tic disorders, ADHD, disruptive disorders, psychotic disorders, eating disorders and pervasive developmental disorders. The available Swedish translation is based on DSM-IV and ICD-10. The instrument screens for 24 DSM-IV and ICD-10 psychiatric disorders and suicidality. According to previous studies the interview takes about 30 min to complete after training and sufficient basic knowledge in psychiatric disorders. It is supposed to be sufficient as a short yet reliable interview that can be used both for research and clinical purposes [5]. The interview often takes place with both parent and child present at the same time, but it can also be done with the young person separately. In addition to the standard version of MINI-KID, the MINI-KID-P version was used. This version is rather recently translated into Swedish and is used when the parent is the main respondent. MINI KID-P is recommended when the child is between 4 and 11 years old, but for children between 8 and 11 years there is a choice between the two versions depending on the child’s developmental level. In the text we use MINI-KID as the general designation for the SDI, MINI-KID-S and MINI-KID-P when specifically describing the standard or parent version.

The message at the project-specific education of the interviewers was that the structure of MINI-KID had to be followed: 1) All modules should be presented in order as well as the screening and follow-up questions. 2) The wording of the questions should be followed but sometimes reformulations and clarifications could be made. 3) It was not permitted to add new questions. Following suggestions by Sheehan [5] it was recommended that the interview in most cases should be administered with the child/adolescent and parent(s) together. Separate interviews with the informants were not performed. An instruction specific for the present study was made concerning younger children where some screening questions (alcohol, addiction and suicide) were reworded to be more appropriate for this age group. The interviewers were also given the freedom to stop posing questions when they thought they had enough information (although the instruction according to MINI-KID is to ask all follow-up questions for scoring if screening is positive). After an assessment of the answers, the interviewer using paper/pencil completed the form with diagnoses on the first pages in the MINI-KID questionnaire.

#### Lead

The LEAD assessments were performed by six clinical senior experts, psychiatrists and psychologists with many years of clinical experience and profound knowledge of the diagnostic procedure using DSM-IV (Mean 24.2 years, Standard deviation (SD) 6.6). The experts were trained by making LEAD assessments based on the medical records for three patients. Prior to this procedure, the LEAD protocol had been scrutinized and revised during several meetings with the LEAD assessors. Then LEAD assessment was made six months after the patient’s first visit to the child and adolescent psychiatric outpatient clinic. In the assessment, two clinical experts reviewed all data in the patient’s medical record. In the first step, the diagnoses at the initial assessment were extracted from the medical record. In the second step, the LEAD assessors scrutinized the complete medical record data spanning 6 months. The assessment was meant to capture the diagnoses that best represent the 6-month period. Independently of each other, the assessors assigned the most appropriate DSM-IV diagnoses, followed by a discussion and further scrutinizing of the data until there was consensus concerning the diagnosis/diagnoses. The idea was to have meetings with all LEAD assessors in case non-consensus. However, consensus was achieved in all cases. Thus, the patients were followed up in the medical records spanning 6 months. During that period, the total number of visits after the first visit to the unit was on average 7.5 visits (median 7, SD 4.3, min 2, max 21, the 25th percentile was 4 visits). There was an ongoing contact with the unit after 6 months in 65% of the patients. Out of these, 53.1% were still in a state of assessment or on waiting list for neuropsychiatric assessment and 46.9% were receiving treatment. The average duration of contact with the unit was 3.4 months for those patients who finished the contact before 6 months. The documentation in the medical records serving as a base for the clinical diagnostic procedure was rated by the LEAD-experts. The documentation was very well founded in 15.5%, rather well founded in 54.6%, quite unfounded in 20.6% and very unfounded in 9.3%. “Very unfounded” was defined as medical records with very sparse information as a base for the clinical diagnostic procedure and consequently also for the LEAD procedure. The initial clinical assessment and LEAD assessments resulted in some instances of diagnoses not covered by MINI-KID. This is described in Table 1.

**Table 1:** Syndromal diagnoses according the MINI-KID interview, initial assessment and LEAD assessment

Disorder	MINI-KID interview	Initial assessment	LEAD assessment
	N (%)	N (%)	N (%)
Anxiety disorders	57 (30.5)	23 (24.5)	29 (21.2)
ADHD	37 (19.8)	21 (22.3)	30 (21.9)
Behavioural/disruptive disorders	27 (14.4)	6 (6.4)	7 (5.1)
Depressive disorders	25 (13.4)	15 (16.0)	15 (11.0)
OCD	12 (6.4)	3 (3.2)	7 (5.1)
Pervasive developmental disorders	10 (4.3)	6 (6.4)	15 (11.0)
Tic disorders	9 (4.8)	2 (2.1)	4 (2.9)
Stress disorders	5 (2.1)	5 (5.3)	5 (3.7)
Substance-related disorders	2 (1.6)	3 (3.2)	4 (2.9)
Eating disorders	2 (1.1)	2 (2.1)	2 (1.5)
Bipolar disorder	1 (0.5)	1 (1.1)	1 (0.7)
Learning disorder	–	1 (1.1)	4 (2.9)
Communication disorder	–	2 (2.1)	8 (5.8)
Enuresis	–	1 (1.1)	2 (1.5)
Somatoform disorder	–	1 (1.1)	1 (0.7)
Sleep disorder	–	1 (1.1)	1 (0.7)
Mental retardation, intellectual disability	–	1 (1.1)	1 (0.7)
Total	187(100)	94 (100)	137 (100)

Not included in MINI-KID assessment

### Statistical methods

First a comparison was made between the diagnoses from the MINI-KID disorder classifications and the diagnoses from the regular initial clinical assessment at the outpatient unit. Then the diagnoses in the MINI-KID were compared with the LEAD diagnoses. The most prevalent composite diagnoses according to MINI-KID were used in the comparative analyses; Any anxiety disorder, Any Attention Deficit/Hyperactivity Disorder (ADHD), Any behavioural/disruptive disorder (Oppositional Defiant Disorder (ODD) and or Conduct Disorder (CD)), Any depressive disorder, Any OCD, Any tic disorder and Any pervasive developmental disorder. In addition, “Any diagnosis” was included in the analyses, defined as any of the most prevalent seven diagnoses. Either DSM and ICD-codes or both could be presented in the medical records. The DSM-codes were translated to ICD-10 codes using the mapping tables in the Swedish version of the short version of DSM-5 [29].

The diagnostic accuracy of a test refers, in this case MINI-KID, to the extent of agreement between the outcome of the new test and a reference standard. We compare a new measurement system MINI-KID with a well-known diagnostic system LEAD. However, when comparing the diagnoses from the MINI-KID disorder classifications with LEAD diagnoses this could be regarded as comparing a new test is evaluated by comparison to an imperfect gold-standard [30]. In this situation, according to U.S. Food and Drug Administration (2007), you cannot directly calculate unbiased estimates of sensitivity and specificity [31]. Therefore, the terms sensitivity and specificity are not appropriate to describe the comparative results. Instead, the same numerical calculations are made, but the estimates are called positive percent agreement (PPA) and negative percent agreement (NPA), rather than sensitivity and specificity. This reflects that the estimates are not of accuracy but of agreement of the new test with the non-reference standard. PPA is the proportion of non-reference standard (LEAD) positive patients in whom the new test (MINI-KID) is positive. NPA is the proportion of LEAD negative subjects in whom MINI-KID is negative.

As measures of inter-agreement between the diagnostic procedures/tests we also calculated the following: the overall percent agreement (OPA) (the proportion of subjects in whom the new test (MINI-KID) and the non-reference standard (LEAD) give the same outcome), predictive value of a positive result (PPV) (the proportion MINI-KID positive patients who have the target condition according to LEAD, predictive value of a negative result NPV (the proportion of MINI-KID negative patients who do not have the target condition according to LEAD).

The predictive values described above are not invariant characteristics of the tests and significantly depend on the prevalence of the disease in the population tested. In order to solve this problem, the Likelihood Ratio (LR) was used as a measure which is independent of prevalence. Thus, we also calculated the positive likelihood ratio (PLR) ((1-PPA)/NPA) and negative likelihood ratio (NLR) (PPA/(1-NPA)). However, it should be noted that these calculations were performed using an imperfect reference standard, the research diagnoses according to LEAD. The Likelihood Ratio (LR) is the likelihood that a given test result would be expected in a patient with the target disorder compared to the likelihood that that same result would be expected in a patient without the target disorder. LR is regarded as one of the most clinically useful measures. PLR is usually a number greater than one and the NLR ratio usually is smaller than one. As a guide to interpretation, PLR above 10 are considered to provide strong evidence to rule in a diagnosis, whereas those between 5 to 10 provide moderate evidence, and those between 2 and 5 provide weak evidence. NLR below 0.1 are considered to provide strong evidence to rule out a diagnosis, whereas those between 0.1 and 0.2 provide moderate evidence, and those between 0.2 and 0.5 provide weak evidence [32].

Data analysis was conducted using the statistical software Stata [33].

## Results

The study group was 50 boys, 51 girls, by region (n), Gothenburg (30), Lund (18), Stockholm (53). The patients’ age varied from four to almost 18 years at the time of MINI-KID interview (M 11.4, median 11.3, SD 3.5, min 4.2, max 17,9). The boys were on average 10.4 years (median 9.9, SD 3.4, min 4.4, max 17.7) and the girls 12.5 (median 12.9, SD 3.3, min 4.3, max 17.9).

The contact with the child psychiatric outpatient unit started with the regular initial clinical assessment and during the first 6 weeks, the average number of visits was 1.9 (median 1 SD 1.3, min 1, max 8). Within these 6 weeks the MINI-KID interview was performed parallel to and blind to the initial assessment. Each interviewer (*n* = 17) performed on average MINI-KID-interviews with four patients. (*M* = 5.3, median 4, min 1, max 21). The duration of the interview was on average 46 min. In 88 of 101 interviews at least one parent participated together with the patient. In five of 101 interviews the patient was interviewed alone (median age 15.0 years) and in eight cases of 101 the parent was interviewed alone (median age 6.1 years). MINI-KID-P was used in 86.3% of the interviews for children younger than 11.3 years (the median age in the study sample). MINI-KID-S was used in 86.0% for children older than 11.3 years.

In Table 1 the total number of each syndromal diagnosis according to the MINI-KID disorder classifications, the regular initial clinical assessment and the research diagnoses according to LEAD is presented. The total number of diagnoses according to MINI-KID was almost double in the initial assessment. The MINI-KID interview yielded a higher number of diagnoses especially for anxiety disorders, behavioral/disruptive disorder (ADHD not included), OCD and tic disorders. The number of diagnoses was calculated for patients with any anxiety disorder; the average number of anxiety diagnoses per patient was 1.6 (min 1 max 4) according to the MINI-KID interview and 1.1 (min 1 max 2) according to the regular initial clinical assessment.

Table 2 presents analyses of the agreement between the MINI-KID disorder classifications and the regular initial clinical assessment according to the medical record. The prevalence was statistically significantly higher for each category of diagnosis for the MINI-KID-interview with one exception – pervasive developmental disorder. OPA for the seven most common diagnoses (any diagnosis not included) between MINI-KID and diagnoses according to the regular initial clinical assessment was 80.6%, PPA 24.0 and NPA 95.2. OPA was highest for Tic disorders (91.1) and Pervasive developmental disorders (88.1). PLR varied from 1.2 to 6.5 and NLR from 0.0 to 0.9.

**Table 2:** Agreement between MINI-KID and the initial assessment according to the medical record

Disorder	MINI-KID	Initial clinical assessment	p^a^	MK+	MK+	MK-	MK-	Measures of agreement between MINI-KID and the initial assessment			
	Prevalence (%)	Prevalence (%)		LEAD+	LEAD-	LEAD+	LEAD-	PPA/NPA	PPV/NPV	PLR/NLR	OPA
Any diagnosis	76.2	63.4	**	52	25	12	12	81.3/32.4	12.5/50.0	1.2/0.6	63.4
Any anxiety disorder	34.7	20.8	***	14	21	7	59	66.7/73.8	40.0/89.4	2.5/0.5	72.3
Any ADHD	36.6	20.8	***	15	22	6	58	71.4/72.5	40.5/90.6	2.6/0.4	72.3
Any behavioural/ disruptive disorder	26.7	5.0	***	5	22	0	74	100.0/77.1	18.5/100.0	4.4/0.0	78.2
Any depressive disorder	20.8	10.9	**	6	15	5	75	54.6/83.3	28.6/93.8	3.3/0.5	72.9
Any OCD	11.9	3.0	***	2	10	1	88	66.7/89.8	16.7/98.9	6.5/0.4	89.1
Any tic disorder	8.9	2.0	***	1	8	1	91	50.0/91.2	11/1/98.9	6.2/0.5	91.1
Any pervasive developmental disorder	7.9	5.9	NS^b^	1	7	5	88	16.7/92.6	12.5/94.6	2.3/0.9	88.1

^a^test if there is a difference in the proportions with a diagnosis according to MINIKID and the LEAD assessment. Data are presented as *, **, ***p value < .05, .01, and .001, respectively

^b^Not Statistically Significant (NS)

Table 3 shows the agreement between the MINI-KID disorder classifications and the LEAD assessment. OPA between MINI-KID and LEAD was 79.5%, PPA 35.4 and NPA 92.7. The average OPA was highest for OCD (0.89), Tic disorders (0.88) and Pervasive developmental disorders (0.81). PLR varied from 1.6 to 9.6 and NLR from 0.3 to 0.8.

**Table 3:** Agreement between MINI-KID and the LEAD assessment

Disorder	MINI-KID	LEAD assessment	p1	MK+	MK+	MK-	MK-	Measures of agreement between MINI-KID and LEAD			
	Prevalence (%)	Prevalence (%)		LEAD+	LEAD-	LEAD+	LEAD-	PPA/NPA	PPV/NPV	PLR/NLR	OPA
Any diagnosis	76.2	78.2	NS	66	11	13	11	83.5/50.0	85.7/45.8	1.7/0.3	76.2
Any anxiety disorder	34.7	18.8	***	12	23	7	59	63.2/72.0	34.3/89.4	2.3/0.5	70.3
Any ADHD	36.6	28.7	NS	20	17	9	55	69.0/76.4	54.1/85.9	2.9/0.4	74.3
Any behavioural/ disruptive disorder	26.7	6.9	***	5	22	2	72	71.4/76.6	18.5/97.3	3.1/0.4	76.2
Any depressive disorder	20.8	18.8	NS	8	13	8	75	61.5/85.2	38.1/93.8	4.2/0.5	71.7
Any OCD	11.9	6.9	*	5	7	2	87	71.4/92.6	41.7/97.8	9.6/0.3	91.1
Any tic disorder	8.9	4.0	*	1	3	8	89	25.0/91.8	11.1/96.7	3.0/0.8	87.8
Any pervasive developmental disorder	7.9	14.9	*	4	4	11	82	26.7/95.4	50.0/88.2	1.6/0.6	85.2

p1 - test if there is a difference in the proportions with a diagnosis according to MINIKID and the LEAD assessment. Data are presented as *, **, ***p value < .05, .01, and .001, respectively

Table 4 shows measures of agreement between MINI-KID disorder classifications and the research diagnoses according to LEAD calculated in two different groups of patients, the MINI-KID-S group (49 patients) and MINI-KID-P group (51 patients). There were no statistically significant differences for any of the diagnoses comparing MINI-KID-S and MINI-KID-P with respect to OPA.

**Table 4:** Measures of agreement between MINI-KID and the LEAD assessment by MINI-KID version

Diagnosis	Measures of agreement between MINI-KID and LEAD					
	MINI-KID-S (*n* = 49)			MINI-KID-P (*n* = 51)		
	PPA/NPA	PLR/NLR	OPA	PPA/NPA	PLR/NLR	OPA
Any diagnosis	84.2/45.5	1.5/0.3	75.5	82.9/50.0	1.6/0.3	76.5
Any anxiety disorder	66.7/73.5	2.5/0.5	71.4	50.0/70.2	1.7/0.7	68.6
Any ADHD	55.6/77.5	2.5/0.6	73.5	75.0/74.2	2.9/0.3	74.5
Any behavioural/ disruptive disorder	100/82.6	5.8/0.0	83.7	50.0/70.2	1.7/0.7	68.6
Any depressive disorder	66.7/77.5	3.0/0.4	75.5	50.0/91.5	5.9/0.5	88.2
Any OCD	100/88.9	9/0	89.8	33.3/95.8	8.0/0.7	92.2
Any tic disorder	100/100	82.8/0.1^a^	91.8	25/91.5	2.9/0.8	86.3
Any pervasive developmental disorder	16.7/95.3	3.6/0.9	85.7	33.3/95.2	7.0/0.7	84.3

^a^LR for tic disorders in MINI-KID-S were estimated using a substitution formula: 0.5 is added to all cell frequencies before calculation

There were no statistically significant differences in the proportions (overall percent agreement) tested for each of the diagnoses between MINI-KID-S and MINI-KID-P (prtesti in Stata)

## Discussion

This study provides additional information on the diagnostic properties of the MINI-KID disorder classifications. We present data from a Child and Adolescent outpatient clinical sample aged 3.5 to 17.9 years (*n* = 101) describing the concordance between the Swedish language version of the MINI-KID Interview and LEAD research diagnoses in secondary child and adolescent psychiatric outpatient care. There was acceptable agreement between MINI-KID disorder classifications and LEAD showing an average overall percent agreement 79.5 between MINI-KID and LEAD, PPA 35.4, and NPA 92.7. According PLR MINI-KID provided moderate evidence to rule in OCD (PLR 9.6) and weaker evidence to rule in depressive disorder, behavioural/disruptive disorder, tic disorder, ADHD and anxiety disorder (PLR:s from 4.2 to 2.3).

We also present measures of agreement between diagnoses from the MINI-KID disorder classifications with the regular initial clinical assessment. There was similar agreement between MINI-KID disorder classifications and the regular initial assessment with regard to OPA 80.6%, PPA 24.0% and NPA 95.2%.

In the study by Sheehan et al. [5] the measures of agreement between MINI-KID disorder classifications and K-SADS-PL were generally somewhat higher than we have found comparing MINI-KID with an initial clinical assessment and LEAD after 6 months. The main question about true validity was put forward by Sheehan et al.: “How well does the MINI-KID, or for that matter other instruments like the K-SADS-PL, actually identify “true” cases in the child and adolescent population?” [5]. We have tried to come closer to true diagnoses using expert clinical opinion based on information from medical records, to generate best estimate diagnoses operationalized by Spitzer’s (1983) LEAD (Longitudinal, Expert, and All Data) Standard [17].

Validity represent the extent to which an instrument is actually measuring what it is intending to measure. Reliability is required as a base for validity, and is the ability of an instrument to differentiate between individuals. The review by Duncan et al. showed a pooled test-retest reliability with Kappa 0.58 and highly variable. Reliability varied across psychiatric disorders and informants [16]. The relatively low levels of agreement in the present study between MINI-KID disorder classifications and LEAD could depend on measurement errors and low reliability associated with the implementation of MINI-KID in the present study. The results could also be an example of a general SDI limitation in clinical and research contexts [16].

The two versions of MINI-KID were compared and their accuracy was similar. The versions were used in two separate groups, the MINI-KID-S group (49 patients) and MINI-KID-P group (51 patients). As recommended by Sheehan [5], the interview was administered with the child/adolescent together with the parent(s) in most cases. In a few interviews the teenager was interviewed alone and the parent alone for a few younger children about 6 years of age. MINI KID-P is recommended when the child is between 4 and 11 years old, but for children between 8 and 11 years there is a choice between the two versions depending on the child’s developmental level. We found no statistically significant differences tested for each of the diagnoses in the proportions (percent agreement) between MINI-KID-S version for children/adolescents and the parent version of MINI-KID-P.

In the psychometric evaluation by Duncan et al. [12] the adolescents and parent were interviewed separately, administering the MINI-KID-S to adolescents and the MINI-KID-P to parents. They found generally low agreement between parent and adolescent classification of disorders. On one hand, it could be an effective way to have both the parent and the child/adolescent in the room to get their views at the same time. On the other hand, in some cases it could be difficult for a teenager to tell everything when together with a parent. Thus, MINI-KID has been used in somewhat different ways in the recent studies. Another example is the epidemiological study by Li et al. [34] using MINI-KID-P where only the parent or guardian was interviewed.

The main aim of the present study was to evaluate the diagnostic properties of MINI-KID disorder classifications in comparison with LEAD. In order to avoid bias in the estimates of the concordance between the results of the MINI-KID Interview and the research diagnoses according to LEAD, the MINI-KID interview was done separately from the regular initial clinical assessment and the following clinical work-up. No information of the results of MINI-KID were included in the LEAD assessment. This is in contrast to other validity studies of standardized diagnostic interviews including the results of the interview in focus for the study when performing the LEAD assessment for example KSADS [14].

As expected, MINI-KID disorder classifications doubled in the total number of preliminary diagnoses compared to the regular initial clinical assessment. It is arguable that many of these diagnoses from MINI-KID were not actually false. The regular initial clinical assessment could miss diagnostic information and decisions on a diagnosis by not collecting all relevant data or terminating the interview before exploring all alternatives [3]. According to our clinical experience, many clinicians are hesitant to suggest diagnoses at an initial phase of the diagnostic procedure. The phase of assessment seemed also very extended since many patients in our study were still in a state of assessment or on waiting list for further assessment even after 6 months. This resulted in a problem with the LEAD assessments since some of the medical records, at least in 9.3%, had very sparse information as a base for the LEAD assessment.

The MINI-KID interview resulted in a higher number of anxiety disorders, up to four separate anxiety diagnoses, compared with the regular initial clinical assessment of up to two diagnoses. In addition, the difference in the number of diagnoses were pronounced for behavioural/ disruptive disorder, OCD and Tic disorder. It seems probable that the specific screening questions in MINI-KID reveal important problems at an early phase of the assessment, that may not be asked about or documented in the regular initial clinical assessment. These problems were not always found later on in in the assessment according to LEAD. Nevertheless, the difference in the number between MINI-KID disorder classifications and LEAD diagnoses were less pronounced than between MINI-KID and the initial clinical assessment.

There were on average almost two (1.9) visits during the regular initial clinical assessment during the first 6 weeks but for many patients several more visits. A visit to the outpatient clinics is most often scheduled to 45–60 min. The MINI-KID interview took only about 30 min to complete in the study by Sheehan et al. [5] and in our study somewhat longer, on average 46 min. It could to be time-efficient to use MINI-KID at the initial clinical assessment in child and adolescent care similar to the setting in the present study. It does not take long time and gives information having the potential to be useful in the diagnostic process.

This study has several strengths: it includes an evaluation of the two versions of MINI-KID, MINI-KID-S for youth and parents MINI-KID-P in a secondary level outpatient child and adolescent psychiatry setting. The validity of the most prevalent MINI-KID diagnoses was calculated based on comparisons with LEAD after 6 months’ follow-up as the criterion. The MINI-KID disorder classifications were also compared with the initial clinical diagnoses. The design included blindness between MINI-KID and the other two measures, LEAD and the initial clinical diagnosis. This made it possible to study the MINI-KID results per se in a non-biased way. Several clinicians performed the interviews after training and the situation was very similar with a natural clinical setting and this avoided bias compared to a situation with very few research-interviewers.

There are limitations as well: A larger sample size would have made it possible to study a higher number of the diagnoses included in MINI-KID and not only the seven most prevalent diagnoses. There are also several levels in the data: the patients/the interviewers/the clinics/the three regions. Due to the limited sample size (*n* = 101) it was considered not meaningful to adjust the statistical calculations with regard to the hierarchical structure of the data. The reason for the longer period of data collection, than we previously expected, was that generally a very small proportion of the families in the target population were asked by the staff / accepted to participate in the study, given that for example the three clinics in Stockholm had 11,409 new referrals during 2013–2016. There were also logistic difficulties performing the MINI-KID interviews within the time limit (not more than 6 weeks after the first regular visit to the outpatient unit) for the families who were willing to participate in the study. The blindness between MINI-KID and LEAD could be looked upon as a strength in the design but it also caused a limitation for the LEAD assessment. LEAD had probably come closer to “true” diagnoses if based on all available data.

## Conclusions

There was an overall acceptable agreement between MINI-KID disorder classifications and research diagnoses according to LEAD. The two versions MINI-KID-S and MINI-KID-P had a similar accuracy. MINI-KID-P could be recommended when the child is between 4 and 11 years old, but for children between 8 and 11 years there is a choice between the two versions depending on child’s developmental level. The specific screening questions in MINI-KID resulted in additional preliminary diagnoses compared with the regular initial clinical assessment. It seems probable that the specific screening questions in MINI-KID reveal important problems at an early phase of the assessment. The standardized interview MINI-KID could be considered as a tool with the potential to give valuable information in the diagnostic process in child and adolescent care similar to the setting in the present study.

## Abbreviations

ADHD: Attention Deficit/Hyperactivity Disorder
BCFPI: Brief Child and Family Phone Interview
CD: Conduct Disorder
DSM: Diagnostic and Statistical Manual of Mental Disorders
ICD: International Statistical Classification of Diseases and Related Health Problems
K-SADS-PL: Schedule for Affective Disorders and Schizophrenia for School Aged Children-Present and Lifetime Version
LEAD: Longitudinal, Expert, All Data
LR: Likelihood Ratio
MINI: Mini-International Neuropsychiatric Interview
MINI-KID: The Mini International Neuropsychiatric Interview for Children and Adolescents
MINI-KID-P: Parent version of MINI-KID
MINI-KID-S: Standard version of MINI-KID
NLR: Negative likelihood ratio
NPA: Average percent negative agreement
NPV: Predictive value of a negative result
NS: Not Statistically Significant
OCD: Obsessive-Compulsive Disorder
ODD: Oppositional Defiant Disorder
OPA: Average overall percent agreement
PLR: Positive likelihood ratio
PPA: Average percent positive agreement
PPV: Predictive value of a positive result
SD: Standard deviation
SDI: Structured diagnostic interviews
